# Simultaneous Determination of Multiple Contaminants in Chicken Liver Using Dispersive Liquid-Liquid Microextraction (DLLME) Detected by LC-HRMS/MS

**DOI:** 10.3390/foods12132594

**Published:** 2023-07-04

**Authors:** Belete Eshetu Gebreyohannes, Simiso Dube, Mathew Muzi Nindi

**Affiliations:** 1 Department of Chemistry, The Science Campus, College of Science Engineering and Technology, University of South Africa, Corner of Christiaan de Wet Road & Pioneer Avenue, Florida 1709, South Africa; 48221783@mylife.unisa.ac.za (B.E.G.); dubes@unisa.ac.za (S.D.); 2 Institute for Nanotechnology and Water Sustainability, The Science Campus, College of Science Engineering and Technology, University of South Africa, Corner of Christiaan de Wet Road & Pioneer Avenue, Florida 1709, South Africa

**Keywords:** pesticides, sulphonamides, fluoroquinolones, anthelmintics, Aflatoxin B1, dispersive liquid-liquid microextraction, biological samples, solid phase extraction

## Abstract

Simultaneous determination of a mixture of food contaminants, including pesticides, sulphonamides, fluoroquinolones, anthelmintics, and aflatoxin B1, in solid biological samples (chicken liver) by dispersive liquid-liquid microextraction/liquid chromatography-high resolution mass spectrometry (DLLME/LC-HRMS) is presented. Previous work focused on the application of DLLME to single-class contaminants. In this work, the DLLME extraction method has been extended to complex multiresidues in the biological matrix. The first part of this study was the selection of an appropriate solvent that enabled the dissolution of analytes from the chicken livers. The matrix-matched calibration curves showed good linearity in the range 0.5–50.0 µg kg^−1^ for aflatoxin B1 and 50–500 µg kg^−1^ for pesticides, fluoroquinolones, sulphonamides, and anthelmintics, with a coefficient of determination (R^2^) values of 0.9916–0.9967. The mean recoveries were in the range of 80.4–96.3%, and the relative standard deviation (RSD) values were in the range of 1.53–8.98%. The limit of detection (LOD) and the limit of quantification (LOQ) values were 0.03 µg kg^−1^ and 0.09 µg kg^−1^, respectively, for aflatoxin B1, and for pesticides, fluoroquinolones, sulphonamides, and anthelmintics, they were in the range of 0.011–1.197 µg kg^−1^ and 0.150–2.579 µg kg^−1^, respectively. The developed method was compared with the standard solid phase extraction (SPE) method, and there was no significant difference between the two methods.

## 1. Introduction

The safety of food is important in our everyday lives because of its impact on our health. Globally, an increase in scientific knowledge on chemical contamination of food destined for human consumption has been observed. The concern for food safety is also of great importance to world trade due to public health. Billions of people in the world are at risk from unsafe food due to physical, chemical, and biological contamination, especially organic contaminants such as pesticides, veterinary drugs, persistent environmental chemicals, and naturally occurring toxicants [1,2]. Contamination of foods can occur during production [3], storage, transportation, food processing [4], and the use of veterinary drugs in food-producing animals [5]. In addition, the safety and quality of food products have become a growing concern for consumers, governments, and producers because the presence of unwanted and/or threatening contaminants in foods such as meat, animal products, etc. impacts both the local and export economies [6,7,8]. Several institutions worldwide have put systems in place to protect humans from exposure to many of the chemicals identified as unsuitable for human consumption. The European Union (EU), the United States Environmental Protection Agency (USEPA), the United States Food and Drug Administration (FDA), Codex Alimentarius (Codex Alimentarius Commission, FAO-WHO), as well as other public health agencies around the world, have set maximum residue limits for contaminants in animal food and products. As an example, the use of veterinary drugs in the EU is regulated through Council Regulation 2377/90/EC (European Commission (EC) 2002). In general, regulatory bodies have set maximum residue limits (MRLs) for mycotoxins, veterinary drugs, and pesticides in products of animal or vegetable origin that are intended for human or animal consumption in order to assure human food safety (Table 1).

Therefore, in order to control and monitor contaminants in food, analytical methodologies must accurately identify and quantify the occurrence of harmful chemical substances in food samples. Sample preparation steps are key to any analytical methodology, as reflected by the time and cost of this step. The primary goal of any extraction technique is the isolation and/or preconcentration of analytes of interest from the complex sample matrix. In most cases, it is difficult, if not impossible, to determine analytes of interest from the food sample directly without sample preparation methods [12]. Some methods for comprehensive analysis of harmful chemicals (mycotoxins, veterinary medications, and pesticides) have been developed; however, they are time-consuming, expensive, and harmful to the environment [12,13,14,15,16,17]. In recent decades, researchers have focused on the miniaturisation of extraction techniques and the use of solvents that are less harmful to the environment (green solvents) in an attempt to address environmental issues as well as challenges associated with biological matrices. These new analytical techniques are compliant with green analytical chemistry principles [18,19]. One such method that has drawn the interest of many researchers is dispersive liquid microextraction due to its advantages of being simple, cheap, rapid, green, and having high efficiencies. This miniaturized sample preparation method is attractive since it uses microlitre volumes of organic solvents yet is capable of achieving high enrichment factors and producing clean extracts. In the past, the approach to residue analysis has targeted single-class organic contaminants in food. For example, a number of DLLME applications in biological matrices are targeted at single-class contaminants using different analytical instrumentation. Deng et al. [20] extracted sulphonamides, while Moema et al. [21] extracted fluoroquinolones from chicken livers using DLLME. In another research work, Liu and co-workers [22] extracted clenbuterol from porcine tissues, and Vinas et al. [23] also reported the extraction of thiamines from foods using DLLME. However, the continuous detection of various types of contaminants in food necessitates the development of analytical methods that can handle multiclass residues. This current work extends the capabilities of DLLME to a mixture of various classes of contaminants in response to recent developments in FAO and Codex.

In this work, a rapid, cheap, simple, and green extraction method, DLLME, was developed and validated for the simultaneous determination of multiple contaminants in chicken liver samples using LC MS/MS. To the best of our knowledge, this is the first DLLME method reported for the simultaneous determination of multiple contaminants like aflatoxin B1, pesticides, fluoroquinolones, sulphonamides, and anthelmintics in biological chicken matrices.

## 2. Experimental

### 2.1. Chemicals, Reagents, and Materials

All reagents and solvents were analytical and LC-MS grade. The following individual standards (>97% purity) and internal standards (IS) used in this study were purchased from Sigma-Aldrich (Steinheim, Germany): aflatoxin B1, albendazole, atrazine, danofloxacin, enrofloxacin, fenbendazole, mebendazole, simazine, sulphachloropyridazine, sulphadiazine, sulphamerazine, sulphaquinoxaline, sulphapyridine, terbutryn, thiabendazole, Aflatoxin B1-13C17 (IS), Albendazole-d3 (IS), Atrazine-d5 (IS), Danofloxacin-(methyl-d3) (IS), Enrofloxacin-d5 (IS), Fenbendazole-d3 (IS), Mebendazole-d3 (IS), Simazine-d10 (IS), Sulfamethazine-d4 (IS), Sulfaquinoxaline-d4 (IS), Sulfapyridine-d4, Terbutryn-d5 (IS). Reagents used in the experiments were HPLC and LC-MS-grade solvents and were purchased from Sigma Aldrich (Steinheim, Germany). These include acetone, chlorobenzene (C_6_H_5_Cl), 1,1,2,2-tetrachloroethane (C_2_Cl_4_), HPLC grade, while methanol, acetonitrile (LC-MS grade), and formic acid were purchased from Romil Ltd. (Cambridge, UK). Ultrapure water (18.2 MΩ cm at 25 °C) was processed by the Milli-Q^®^ Reference Water Purification System (Merck Millipore, Bedford, MA, USA). Nitrogen gas (N_2_) of 99.9% purity was generated using a Genius 1022 Nitrogen Generator (Peak Scientific Inc., Billerica, MA, USA). The Pierce™ Calibration Solutions (Pierce™ ESI Negative Ion Calibration Solution and Pierce™ LTQ ESI Positive Ion Calibration Solution) (10 mL) were obtained from Thermo Fisher Scientific (Rockford, IL, USA). Table S1 lists the structures, CAS numbers, pKa values, and K_ow_ values (listed in the table as log P) of target analytes used in this study.

### 2.2. Preparation of Standard Solutions

Individual stock solutions of 1000 µg mL^−1^ of aflatoxin B1, SQ, SPD, SDZ, SCP, SMZ, DFX, ENR [24,25,26], SIMZ, TRB, ATR, TBZ, FBZ, ABZ, and MBZ were prepared by accurately weighing out standards using a Mettler Toledo XP6U Micro Comparator balance (Greifensee, Switzerland) and dissolving them in either ACN, MeOH, water, or dimethyl sulfoxide depending on the solubility of each compound. Isotopically labelled internal standards (IS) solutions of aflatoxin B1-13C17, Albendazole-d3, Atrazine-d5, Danofloxacin-(methyl-d3), Enrofloxacin-d5, Fenbendazole-d3, Mebendazole-d3, Simazine-d10, Sulfamethazine-d4, Sulfaquinoxaline-d4, Sulfapyridine-d4, Terbutryn-d5 were prepared separately but in the same way as the working standard solution mixture. The stock solutions of the individual standards were used to prepare working mixture solutions and calibration standard solutions.

Matrix-matched calibration standard solutions were prepared by spiking the blank liver samples with appropriate volumes of the target analyte working standards such that the concentration range was 5 to 500 µg kg^−1^ for pesticides, sulphonamides, fluoroquinolones, and anthelmintics and 0.5 to 50 µg kg^−1^ for aflatoxin. This was followed by adding isotope-labelled standards to each matrix matched calibration standard sample. The matrix-matched calibration standard samples were then treated with the DLLME procedure as described in Section 2.4 and Section 2.5 below. The standard solutions were stored at a temperature of 2–8 °C until ready for analysis.

### 2.3. LC-HRMS Analysis

#### 2.3.1. Mass Spectrometry

A Thermo Scientific™ Q Exactive™ Plus Hybrid Quadrupole-Orbitrap™ Mass Spectrometer coupled to a Thermo Scientific™ Dionex UltiMate™3000 UHPLC system (Thermo Fisher Scientific, Waltham, MA, USA) was used in this study. Detection was carried out using Exactive™ Plus LC-MS/MS equipped with a heated electrospray ionisation (HESI) probe in multiple reaction monitoring (MRM) mode. The optimum ion source conditions compatible with the HPLC flow rate were used (capillary temperature of 290 °C; sheath gas flow, 50 arbitrary units (AU); spray voltage, 3.5 kV; auxiliary temperature, 400 °C). Analysis was performed in full MS, single ion monitoring (SIM) mode, and all-ion fragmentation (AIF) in positive ion mode over a scan range from *m*/*z* 80 to 750 with a mass accuracy of <5 ppm. The mass spectrometer was operated at a mass resolution offset of 70,000 full width at half maximum (FWHM), with the automatic gain control (AGC) target set at 1.0 × 10^6^ and a maximum injection time (IT) of 100 ms. The mass spectrometer was calibrated weekly for mass accuracy using Thermo Scientific™ Pierce™ Calibration Solutions (Pierce™ ESI Negative Ion Calibration Solution and Pierce™ LTQ ESI Positive Ion Calibration Solution).

#### 2.3.2. HPLC Separation

A Waters^®^ XBridge™ C18 (3.5 µm, 4.6 × 75 mm) column was used, with 0.1% (*v*/*v*) formic acid in water as mobile phase A and 0.1% (*v*/*v*) formic acid in acetonitrile as mobile phase B. Linear gradient elution was used for the separation, starting from 2% to 45% B in 6 min, then held for 0.5 min, and decreased again to 2% B in 1.5 min, followed by a re-equilibration time of 1 min for the next run; the total run time was 8 min, at a flow rate of 0.5 mL min^−1^, a sample injection volume of 10 µL, and the column temperature was set at 35 °C. The data analysis and processing were carried out using the Qual/Quan Browser TraceFinder software package (TraceFinder, Thermo Fisher Scientific, Waltham, MA, USA).

### 2.4. Sampling and Sample Pre-Treatment

The chicken liver biological matrix was utilised for method optimisation and validation and was acquired from local supermarkets in Gauteng province, South Africa. In this study, chicken liver blank samples were obtained from non-commercial, informal organic farmers. Blank samples were screened and confirmed to be free of residues of the analytes of interest (fluoroquinolones, pesticides, sulphonamides, anthelmintics, and aflatoxin B1). The samples were wrapped in aluminium foil to protect them from photo-degradation and then stored at 4–8 °C. The procedure for the extraction of the analytes of interest from chicken livers consisted of sample pre-treatment and DLLME. The pre-treatment procedure was performed as reported by Moema et al. [21], with some modifications. Chicken livers were chopped up into smaller pieces and homogenised, using a food processor to produce a puree. Homogenised liver samples, i.e., 5 g of the homogenate were weighed into 50 mL Falcon tubes. The blank chicken liver samples were spiked with standard solutions and internal standard solutions of analytes (fluoroquinolones, pesticides, sulphonamides, anthelmintics, and aflatoxin B1). The samples were then treated with 5 mL of 0.08% HF:MeCN (15:85) and mixed on a vortex mixer for 30 s, then centrifuged for 10 min at 4000 rpm. Optimisation studies were carried out using various concentrations of formic acid. The supernatant (acetonitrile extract) was transferred into a vial, and a 1 mL aliquot of the acetonitrile was used for the DLLME procedure.

### 2.5. Dispersive Liquid-Liquid Microextraction

For the DLLME extraction procedure, 5 mL of UHP water was first placed into a 15 mL centrifuge tube (Figure S1). Thereafter, a mixture of 1 mL of the acetonitrile sample extract (used as a disperser solvent) obtained from the earlier procedure and 400 μL of tetrachloroethane (the extraction solvent) were rapidly injected into the tube using a 5 mL syringe with a sharp needle to induce the formation of a cloudy solution that results from the dispersion of fine droplets of the extraction solvent in the water sample. The content was then centrifuged at 4000 rpm for 10 min to separate the organic phase (sedimented bottom layer) from the aqueous phase (upper layer). The lower/organic phase was withdrawn with a microsyringe and transferred into a 1.5 mL vial for evaporation of the solvent under nitrogen. The residue was reconstituted in 500 μL of the mobile phase and injected into the LC-MS/MS for analysis.

### 2.6. Solid Phase Extraction

A solid-phase extraction technique was adopted by Zhang et al. (2018) with some modifications [24]. A chicken liver sample (5.0 ± 0.01 g) was weighed into a 50-mL conical centrifuge tube. An amount of 5 mL of 0.08% formic acid: MeCN (15:85) was added and the tube was vortexed for 10 s. The sample was sonicated for about 30 min, then centrifuged at 4000 rpm for 20 min. The resulting supernatant solution was slowly transferred into a 15-mL centrifuge tube. A Waters Oasis HLB cartridge (12 cc, 500 mg) was set up for pass-through filtration. Before extraction, each Waters HLB cartridge was pre-conditioned with 3 mL of methanol and then rinsed with 3 mL of deionized water on an SPE manifold. An extract sample was then passed through the HLB cartridge. After extraction, the cartridge was washed with 1 mL of 5% methanol in water and subsequently air-dried under a vacuum for at least 20 min. The residues were then eluted from the cartridge with 2 portions of 5 mL of MeCN (LC grade). All the extracts were completely evaporated to dryness by a gentle stream of nitrogen. The dried sample under a gentle stream of nitrogen was followed by reconstitution in 500 μL of acetonitrile, and 10-μL aliquots were injected into HR-LC MS/MS system.

## 3. Results and Discussion

### 3.1. Mass Spectrometry Optimisation

The mass spectrometry (MS) method development was conducted through the introduction of target analytes into the mass spectrometry system via direct infusion of the standard solutions. All the target analytes and internal standards were found to be more sensitive in the positive ion mode. Previous studies confirmed the presence of the precursor ion [M+H]^+^ of aflatoxin B1 [25], sulphonamides (SQ, SPD, SDZ, SCP, and SMZ) [26,27,28,29,30], fluoroquinolones (DFX and ENR) [31,32,33], pesticides (SIMZ, TRB, ATR, and TBZ) [34,35], and anthelmintics (FBZ, ABZ, and MBZ) [34,35]. Solutions of individual analytes in acetonitrile containing 0.1% (*v*/*v*) formic acid were infused at a flow rate of 10 µL min^−1^ to determine the MS conditions for each analyte. Multiple reaction monitoring (MRM) was used, and a summary of precursor and product ions and collision energies for each compound is presented in Table 2. The developed separation method (Figure S4) in combination with MRM conditions generated typical ion chromatograms of the mixture of mixed contaminants (Figure S5).

### 3.2. Optimisation of DLLME Conditions

**Figure 1:**
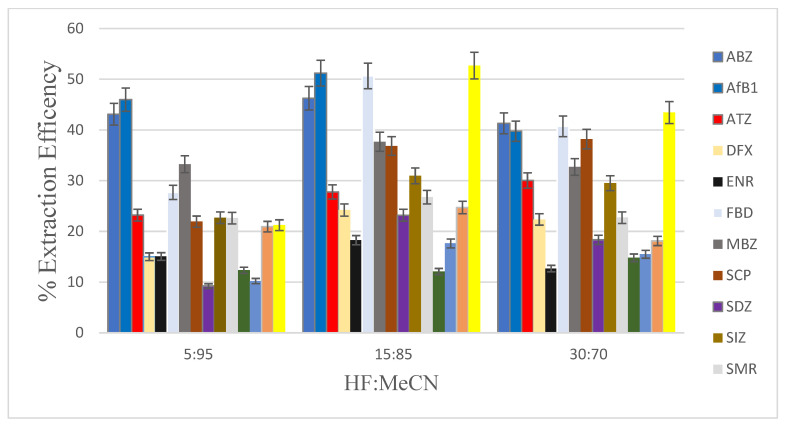
The extraction efficiencies of mixed contaminants (concentration of 100 µg kg^−1^ was used for all sulphonamides, fluoroquinolones, pesticides, anthelmintics; concentration of 10 µg kg^−1^ for aflatoxin) from spiked blank chicken liver biological matrix.

The DLLME extraction method was optimised for aflatoxin B1, pesticides, fluoroquinolones, sulphonamides, and anthelmintics in the chicken livers. The biological matrix extraction method was adopted from Moema et al. [21]. Various parameters that affect extraction efficiency, such as the type and volume of extraction solvents and the type and volume of disperser solvents, and the sample pH, were optimised. In DLLME extraction recovery (ER) was used to evaluate the extraction efficiency to obtain optimized extraction conditions. The extraction recovery was calculated: ER = {C_sed_ × V_sed_}/{V_o_ × V_eq,_}× 100 where: (*C*_sed_) and (*C*_0_) initial concentrations of analytes within the sample and concentrations in the sediment phase, and *V*_sed_ and *V*_aq_ are the volumes of the sediment phase and sample solution, respectively. The blank chicken liver samples were spiked with the analytes at a concentration of 100 µg kg^−1^ for pesticides, fluoroquinolones, sulphonamides, and anthelmintics, and at a concentration of 10 µg kg^−1^ for aflatoxin B1, and treated with 0.08% formic acid in water and acetonitrile (MeCN). The effect of the percent composition of formic acid in water/acetonitrile on the extraction efficiencies for mixed contaminants from blank chicken liver samples was investigated. The best ratio of formic acid in water (HF) and acetonitrile (MeCN) was found to be 15:85 (Figure 1). This solution was further used as the disperser solvent. Five mL of UHP water were placed into the Falcon tube, and the disperser and extraction solvents were rapidly added to the tube. For all the optimisation studies, peak areas were used to evaluate the extraction efficiencies.

#### 3.2.1. Selection of Disperser Solvent

**Figure 2:**
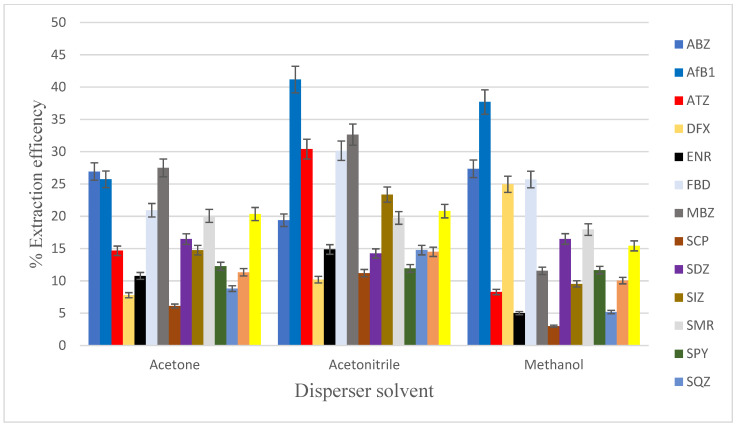
Effect of types of disperser solvent on extraction efficiency in the extraction of mixed contaminants (concentration of 100 µg kg^−1^ for sulphonamides, fluoroquinolones, pesticides, anthelmintics; concentration of 10 µg kg^−1^ for aflatoxin B1) in DLLME. Extraction conditions: 5 mL of UHP water; 400 μL of extraction solvent (tetrachloroethane); 1000 μL of disperser solvent (acetone, acetonitrile, methanol).

The disperser solvent is one of the ternary solvents that plays a key role in the DLLME extraction process, and it is a very important parameter to be optimise. It is a prerequisite that it should be miscible with both the aqueous and organic phases. Additionally, it is necessary that the disperser solvents disperse the extraction solvent into very fine droplets in the aqueous sample to increase contact area; the increased surface area of the droplets assists in the instantaneous partitioning of the analytes from the aqueous phase into the organic phase. The disperser solvent is critical for the formation of cloudiness due to the presence of fine droplets of extraction solvent dispersed throughout the aqueous phase. In this, acetone, acetonitrile, and methanol were evaluated as possible disperser solvents. The results showed the best extraction efficiency from the tested solvents in acetonitrile (Figure 2). Aflatoxin B1 was found to be most amenable to extraction, with recovery efficiencies ranging from 26–41%. This observation was very interesting considering the diversity of the analytes in this work and can be attributed to the high compatibility of acetonitrile with the aqueous solution in comparison with acetone and methanol. Therefore, acetonitrile was selected as the disperser solvent for all further experiments.

#### 3.2.2. Extraction Solvent Selection

**Figure 3:**
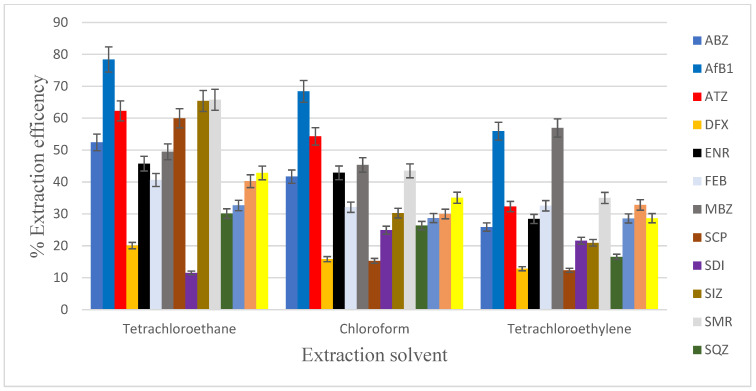
Effect of types of extraction solvent on extraction efficiency in extracting mixed contaminants (concentration of 100 µg kg^−1^ for sulphonamides, fluoroquinolones, pesticides, anthelmintics; concentration of 10 µg kg^−1^ for aflatoxin B1) in DLLME. Extraction conditions: 5 mL of UHP water; 400 μL of extraction solvent (tetrachloroethane); 1000 μL of disperser solvent (acetone, acetonitrile, methanol).

The selection of an appropriate extraction solvent is also important for the successful and efficient use of the DLLME. In the conversional DLLME, the extraction solvent has a density that is higher than that of water [36]. This facilitates the separation via centrifugation of the extractant analyte from the aqueous environment. In addition, the solvent must have good extraction capability for the compounds of interest, good chromatographic behaviour, and miscibility with water [37]. In this study, three organic solvents, with their densities given in parentheses, including tetrachloroethylene (1.62 g cm^−^^3^), chloroform (1.49 g cm^−^^3^), and tetrachloroethane (1.59 g cm^−^^3^), were investigated as potential extraction solvents. Spiked chicken liver biological matrix samples were exposed to these different extraction solvents according to the procedure given in the experimental section (Section 2.5). Figure 3 clearly shows that tetrachloroethane was the most efficient extraction solvent in comparison to the other two solvents. Therefore, tetrachloroethane was used for further extraction work.

#### 3.2.3. Optimisation of pH of the Sample

The pH is important in microextraction techniques, and in this study, pH optimisation was carried out. The distribution ratio of target analytes between the aqueous and organic phases is pH-dependant. Therefore, optimisation of pH is an attempt to get as many of the analytes in complex biological matrices into an extractable form [38,39,40], especially considering the diversity of the analytes of interest in this study. For example, amphoteric sulphonamides, with their pKa values in the pH ranges of 1.97–2.14 and 4.3–6.99, tend to exist as anionic forms in alkaline solutions [39,40]. The quinolone class of antibiotics is also amphoteric, with pKa values ranging from 5.63–6.73 and 5.69–6.68). In addition, multiple forms (cationic, anionic, zwitterionic, and neutral) could be expected in basic donor solutions [40,41]. The target pesticides are also ionisable.

**Figure 4:**
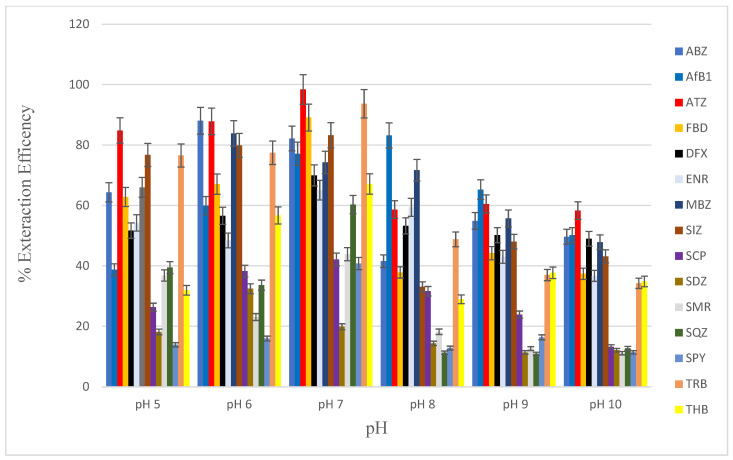
Effect of sample pH on extraction efficiency in extracting mixed contaminants (concentration of 100 µg kg^−1^ for sulphonamides, fluoroquinolones, pesticides, anthelmintics; concentration of 10 µg kg^−1^ for aflatoxin B1) in DLLME. Extraction conditions: 5 mL of UHP water; 400 μL of extraction solvent (tetrachloroethane), 1000 μL of disperser solvent (acetonitrile).

In this study, the effect of sample pH on the extraction efficiencies for the target analytes was evaluated by varying the pH values from 5 to 10 using NaOH and HF (Figure 4). It was observed that all target analytes were extracted above 11% within the pH range of extraction. At pH ranges between 6 and 7, it was observed that most compounds were extracted with efficiencies > 50%. Extraction efficiencies of more than 50% for eight compounds were achieved at pH 6, while 11 compounds were extracted at efficiencies > 60% at pH 7 (Figure 4). The optimum pH for the extraction of analytes of interest was pH 7, where five analytes (ABZ, ATZ, FBD, SIZ, and AFB1) were extracted with efficiencies > 80%. This observation was an indication that the neutral forms of the analytes were more efficiently extractable than their ionic forms. In addition, it appears the stability of the target analytes was maintained in weakly acidic and alkaline environments. The degradation of target analytes in strongly acidic and alkaline conditions that has been suggested [38,39,40] did not impact this study. For the extraction of analytes of interest, the sample pH was therefore maintained at pH 7.

#### 3.2.4. The Effect of Extraction Solvent Volume

The volume of extraction solvent is an important parameter and has a major effect on the extraction efficiency [42,43]. As the extraction solvent volume increased, dilution of the analyte was observed. The extraction efficiency remains constant, resulting in a decrease in the sensitivity of the determination for the target compounds [44,45]. In this work, the effect of extraction solvent volume was investigated by varying the volume from 100 to 500 µL, while maintaining all other parameters constant. The results showed that with an increase in the solvent volume, an increase in the extraction efficiencies was observed, up to a maximum at 400 µL (Figure S2). The results also showed that at volumes >400 µL there was a slight decrease in the extraction efficiencies. The observed decrease was due to the dilution of the analytes caused by the increase in the volume of the organic (sedimented) phase. The extraction efficiencies ranged from 22–95% for all the analytes, with an extraction efficiency of >60% observed for 10 compounds and >78% for eight compounds. Hence, 400 μL of extraction solvent volume was used for all subsequent experiments.

#### 3.2.5. The Effect of Disperser Solvent Volume

The cloudiness of the solution, the degree of dispersion of the extraction solvent in the aqueous phase, and the extraction efficiency are dependent on the disperser solvent volume [44]. The effect of the volume of the disperser solvent, i.e., acetonitrile, on the extraction efficiency was investigated by varying the volume from 250 to 1500 μL, while maintaining all other parameters constant. The results showed an increase in extraction efficiencies was observed with an increase from 500 to 1000 μL, followed by a decrease in extraction efficiencies at volumes >1000 μL (Figure S3). The results showed that extraction efficiencies of 38 to 96% for all analytes were achieved at a MeCN volume of 1000 μL. It was observed that 11 compounds had extraction efficiency >59%, whereas seven compounds had an extraction efficiency >79% [45]. This is because at significantly high volumes, the volume of the sedimented phase is increased, thus lowering the partitioning of the analytes into tetrachloroethane [46]. Similarly, when the disperser solvent volume was too low, the cloudiness was low, and therefore the recovery of analytes was also low. However, when the disperser solvent volume was too high, the solubility of the analytes in the aqueous phase increased, and therefore the extraction efficiency decreased; the decrease in distribution coefficient (*D*) or partition coefficient (*P*) plays a role here. The optimum volume of the disperser solvent was taken to be 1000 μL.

### 3.3. Method Validation

As defined by the ISO/IEC 17025 guideline, validation is “the confirmation by examination and the provision of objective evidence that the particular requirements for a specific intended use are fulfilled” [47]. In-house method validation was performed to investigate the applicability of the proposed method according to the main reference documents, namely Commission Decision (EC) No. 2002/657/EC, SANTE/12682/2019 Guidance Document on analytical quality control and method validation procedures for pesticide residues and analysis in food and feed, and the Eurachem Guide: The Fitness for Purpose of Analytical Methods [48,49,50].

Using the optimum extraction conditions of the developed method, the limits of detection, quantification, linearity, recovery, and precision were investigated using spiked liver samples. The linearity of the method was assessed using nine-point matrix-matched calibration curves. It should be noted that at each point, a maximum of three replicates were used. An internal standard was added to each sample. The blank liver samples were spiked with the target analytes in the range from 5 to 500 µg kg^−1^ for pesticides, sulphonamides, fluoroquinolones, and anthelmintics, and in the range of 0.5 to 50 µg kg^−1^ for aflatoxin. Calibration curves were constructed using an analyte/internal standard peak area ratio vs. concentration of analyte. The least-squares regression equations from the calibration curves were used to calculate the limit of detection (LOD) and limit of quantification (LOQ). LODs and LOQs were calculated from the calibration data and regression statistics using the formulas X_LOD_ = 3 S_y/x_/b and X_LOQ_ = 10 S_y/x_/b, respectively, where Sy/x is the standard error and b is the slope of the regression line [51]. The LODs ranged from 0.03 to 1.197 μg kg^−1^, whereas the LOQs ranged from 0.036 to 2.99 μg kg^−1^. Table 3 shows the calibration curve data, together with the coefficient of determination (R^2^), LODs, and LOQs. The calibration curves gave good linearity, at various ranges, with the coefficient of determination (R^2^) ≥ 0.9916 for all target analytes.

In any extraction method, the recovery is an important parameter to assess its suitability. In this work, three concentration levels ranging from 20, 50, and 200 µg kg^−1^ for fluoroquinolones, pesticides, sulphonamides, and anthelmintics and from 2, 5, and 20 µg kg^−1^ for aflatoxin B1 were used for recovery studies. The precision expressed as a relative standard deviation (RSD) of the method was also calculated at these concentration levels. Five replicates of each sample were analysed within a day (intra-day precision), and the procedure was repeated over three consecutive days (inter-day precision) (Table 4).

### 3.4. Comparison of DLLME with SPE

In this work, a paired *t*-test was used to compare the developed and validated DLLME method with the standard SPE method (Waters Oasis HLB cartridge) to assess if the methods were significantly different. For this purpose, the comparison was done using mean recoveries at three concentration levels of mixed contaminant analytes ranging from 20, 50, and 200 µg kg^−1^ for fluoroquinolones, pesticides, sulphonamides, and anthelmintic, and 2, 5, and 20 µg kg^−1^ for aflatoxin B1. Table 5 shows the results of comparison of recoveries using the two extraction methods.

The calculated t-value for all analytes is less than the t-critical value of 2.45, indicating that the results obtained by DLLME and SPE methods do not differ significantly in terms of accuracy. Furthermore, of the two methods, DLLME has the advantages of very short extraction times and the fact that the extraction equilibrium is attained very quickly (a few seconds) compared to the SPE method. Overall, DLLME has the advantages of being very simple, rapid, inexpensive, easy to use, benign to the environment, and not involving any labour-intensive steps compared to solid phase extraction.

### 3.5. Quantification of Mixed Contaminants

The optimised and validated DLLME method was applied to real samples obtained from several local supermarkets in Gauteng province, South Africa. Processed by DLLME extraction and analytes quantified by high resolution LC-MS/MS as described above (Section 2.3 and Section 2.4).

Table 6 shows a summary of determination of the mixed contaminants in 12 different chicken liver samples. Aflatoxin B1, SCP, and ENR were detected in five of the 12 chicken liver samples assayed. The other mixed contaminants were not detected in any of the samples, which might be due to the fact that the concentrations of target analytes in the samples were below the limit of detection (LOD) and limit of quantification (LOQ). All the mixed contaminants detected were found to be below the stipulated South African MRL range and EU MRL range, which might be an indication that proper withdrawal times were observed by farmers. However, since this was a small sample population, more samples still need to be analysed for confirmation.

## 4. Conclusions

The presented DLLME sample preparation method was successful in efficiently extracting a rather complex mixture of food contaminants, which are usually challenging due to their diverse chemical properties. The method was successfully developed, validated, and applied to a biological food matrix such as chicken livers. The developed method was validated with a coefficient of determination (R^2^) range of 0.9916–0.9967, LOD and LOQ of 0.03 µg kg^−1^ and 0.09 µg kg^−1^, respectively, for aflatoxin B1, and LOD and LOQ for pesticides, fluoroquinolones, sulphonamides, and anthelmintics that ranged from 0.011–1.197 µg kg^−1^ and 0.150–2.579 µg kg^−1^, respectively. The mean recoveries were in the range of 80.4–96.3%, and the relative standard deviations (RSDs) were in the range of 1.53–8.98%. Comparison with standard SPE methods shows that DLLME provides acceptable accuracy and thus could be considered as an alternative fast, simpler, and green method for the extraction of multiclass contaminants in food matrices. Therefore, the newly developed method could be used as a routine method for the determination of mixed contaminants in chicken liver samples due to its advantages over other methods.

## Figures and Tables

**Table 1:** Maximum residue limits (MRLs) set by the European Commission and South Africa for selected pesticides, sulphonamides, fluoroquinolones, anthelmintics, and aflatoxin B1 [9,10,11].

Compounds	Species	Maximum Residue Limit (MRL) (µg kg^−1^)	
		SA	EU
Aflatoxin B1	Chicken liver	-	2
Albendazole	All food-producing animals	5000	
Atrazine	All food-producing animals	-	-
Danofloxacin	Chicken liver	400	400
Enrofloxacin	Chicken liver	50	200
Fenbendazole	All food-producing animals	500	-
Mebendazole	All food-producing animals	-	-
Simazine	All food-producing animals	-	-
Sulphachloropyridazine	All food-producing animals	100	100
Sulphadiazine	All food-producing animals	100	100
Sulphamerazine	All food-producing animals	100	100
Sulphaquinoxaline	All food-producing animals	100	100
Sulphapyridine	All food-producing animals	100	100
Terbutryn	All food-producing animals	-	-
Thiabendazole	All food-producing animals	-	-

-: not stated.

**Table 2:** Multiple reaction monitoring (MRM) conditions.

Analyte	Precursor Ion [M + H]^+^, *m*/*z*	Quantifier Ion, *m*/z	Qualifier Ion, *m*/z	Collision Energies
Aflatoxin B_1_	313.0707	285.0755	269.9604	28
Aflatoxin B1-13C17 (IS)	330.1836	234.0700	191.0152	28
Albendazole	266.0955	234.0702	264.0775	15
Albendazole-d3 (IS)	269.1146	234.0691	191.0152	13
Atrazine	216.1011	216.1017	174.0547	10
Atrazine-d5 (IS)	221.1324	216.1015	221.1328	10
Danofloxacin	358.1562	258.1568	81.01760	25
Danofloxacin-(methyl-d3) (IS)	361.1749	360.1725	316.1826	30
Enrofloxacin	360.1718	245.1725	202.0438	37
Enrofloxacin-d5 (IS)	365.2032	360.1725	202.0438	20
Fenbendazole	300.0801	186.0814	242.1442	10
Fenbendazole-d3 (IS)	303.0989	186.0812	242.1438	11
Mebendazole-d3 (IS)	299.1211	264.0773	265.0799	13
Mebendazole	296.1025	264.0764	296.1035	10
Simazine	202.0854	202.0858	84.0015	10
Simazine-d10 (IS)	212.1482	212.1486	313.0712	25
Sulphachloropyridazine	285.0208	156.0116	108.0448	10
Sulphadiazine	251.0597	251.0603	108.0449	10
Sulphamerazine	265.0754	156.0759	108.0449	13
Sulfamethazine-d4 (IS)	283.1161	283.1167	112.0700	13
Sulphaquinoxaline	301.0754	156.0100	301.0760	10
Sulfaquinoxaline-d4 (IS)	305.1005	305.2044	301.0760	13
Sulphapyridine	250.0645	250.0650	108.0449	10
Sulfapyridine-d4 (IS)	254.0896	254.0894	250.0644	10
Terbutryn	242.1429	186.0804	242.1438	10
Terbutryn-D5 (IS)	247.1748	189.0995	245.1621	10

**Table 3:** Analytical performance parameters for the determination of mixed contaminants in chicken liver samples using DLLME method.

Compound	Linear Range (µg kg^−1^)	R^2^	LOD (µg kg^−1^)	LOQ (µg kg^−1^)
Aflatoxin B1	0.5–50	0.9938	0.026	0.086
Albendazole	5–500	0.9939	0.263	0.876
Atrazine	5–500	0.9967	0.292	0.975
Danofloxacin	5–500	0.9925	0.665	2.172
Enrofloxacin	5–500	0.9916	0.774	2.579
Mebendazole	5–500	0.9926	0.109	0.364
Fenbendazole	5–500	0.9949	0.026	0.085
Simazine	5–500	0.9916	0.011	0.036
Sulphaquinoxaline	5–500	0.9927	0.234	0.779
Sulphadiazine	5–500	0.9916	0.465	1.548
Sulphamerazine	5–500	0.9976	0.166	0.597
Sulphachloropyridazine	5–500	0.9917	1.197	2.99
Sulphapyridine	5–500	0.9925	0.278	0.927
Terbutryn	5–500	0.9916	0.040	0.150
Thiabendazole	5–500	0.9947	0.274	0.913

**Table 4:** Recovery and precision of method to determine mixed contaminants in chicken liver samples spiked at three different concentration levels.

Target Compounds	Added (µg kg^−1^)	Detection	% Recovery	Precision	
				Intra-Day % RSD (*n* = 18)	Inter-Day % RSD (*n* = 54)
Aflatoxin B1	2	1.80	90.00	6.57	5.78
	5	4.70	940.0	7.21	6.23
	20	19.00	95.00	2.38	4.52
Albendazole	20	17.77	88.84	4.75	3.87
	50	44.94	89.88	6.22	7.22
	200	158.80	79.40	2.40	4.89
Atrazine	20	17.40	87.00	2.21	3.87
	50	47.80	95.60	1.77	2.77
	200	174.20	87.10	7.45	8.98
Danofloxacin	20	19.70	98.50	6.66	6.75
	50	43.70	87.40	7.14	4.23
	200	168.60	84.30	2.50	5.52
Enrofloxacin	20	19.45	97.25	4.34	8.23
	50	41.75	83.50	8.82	8.66
	200	176.85	88.43	3.01	6.20
Fenbendazole	20	19.54	97.71	6.23	4.44
	50	19.54	39.09	6.23	6.23
	200	182.59	91.30	3.57	4.66
Mebendazole	20	18.43	92.17	6.34	2.56
	50	47.57	95.13	6.05	7.52
	200	166.70	83.35	5.25	6.47
Simazine	20	17.17	85.83	5.54	6.57
	50	48.12	96.24	5.87	7.21
	200	190.02	95.01	7.25	2.38
Sulphachloropyridazine	20	17.81	89.05	5.42	4.75
	50	44.18	88.37	6.49	6.22
	200	164.39	82.20	4.42	2.40
Sulphadiazine	20	18.36	91.79	2.57	2.21
	50	40.41	80.83	2.45	1.77
	200	182.76	91.38	4.34	9.45
Sulphamerazine	20	17.25	86.25	5.53	6.66
	50	43.45	86.90	8.00	7.14
	200	176.85	88.43	8.61	2.50
Sulphaquinoxaline	20	16.17	80.87	2.81	4.34
	50	40.52	81.04	3.09	8.82
	200	173.57	86.78	2.86	3.01
Sulphapyridine	20	16.87	84.33	4.33	6.23
	50	46.18	92.37	5.93	5.23
	200	162.85	81.43	3.07	3.57
Terbutryn	20	16.16	80.82	1.53	6.34
	50	40.39	80.78	2.30	6.05
	200	167.33	83.66	2.27	6.57
Thiabendazole	20	19.07	95.35	1.85	7.21
	50	44.72	89.44	8.53	5.38
	200	186.82	93.41	4.83	6.52

**Table 5:** Comparison of DLLME with SPE method using paired *t*-test, mean recoveries of each of mixed contaminants were compared at three different concentration levels.

Target Compounds	Standard Deviation (SD)	Standard Error of the Mean (SE)	t-Value	t-Critical (0.05)
Aflatoxin B1	4.44	2.56	1.55	2.45
Albendazole	1.06	0.61	−3.17	2.45
Atrazine	1.31	0.76	−0.64	2.45
Danofloxacin	2.99	1.73	−014	2.45
Enrofloxacin	2.99	1.73	−2.54	2.45
Fenbendazole	2.95	1.71	−0.29	2.45
Mebendazole	2.78	1.61	0.78	2.45
Simazine	6.81	3.93	−0.59	2.45
Sulfachloropyridazine	2.66	1.53	−1.49	2.45
Sulfadiazine	1.67	0.96	2.14	2.45
Sulfamerazine	1.98	1.14	−2.81	2.45
Sulfaquinoxaline	4.02	2.32	2.08	2.45
Sulphapyridine	1.87	1.08	−2.27	2.45
Terbutryn	1.28	0.74	0.73	2.45

**Table 6:** Detection of fluoroquinolones, pesticides, sulphonamides, anthelmintics and aflatoxin B1 in chicken liver samples.

Sample	Analytes														
	AFB1	ABZ	ATZ	DFX	ENR	MEB	FEB	SIZ	SCP	SDZ	SMR	SPD	SQ	TER	TBZ
A	<LOD	<LOD	<LOD	<LOD	<LOD	<LOD	<LOD	<LOD	<LOD	<LOD	<LOD	<LOD	<LOD	<LOD	<LOD
B	<LOD	<LOD	<LOD	<LOD	<LOD	<LOD	<LOD	<LOD	<LOD	<LOD	<LOD	<LOD	<LOD	<LOD	<LOD
C	<LOD	<LOD	<LOD	<LOD	41.02	<LOD	<LOD	<LOD	<LOD	<LOD	<LOD	<LOD	<LOD	<LOD	<LOD
D	0.23	<LOD	<LOD	<LOD	<LOD	<LOD	<LOD	<LOD	92.11	<LOD	<LOD	<LOD	<LOD	<LOD	<LOD
E	<LOD	<LOD	<LOD	<LOD	<LOD	<LOD	<LOD	<LOD	<LOD	<LOD	<LOD	<LOD	<LOD	<LOD	<LOD
F	<LOD	<LOD	<LOD	<LOD	<LOD	<LOD	<LOD	<LOD	26.50	<LOD	<LOD	<LOD	<LOD	<LOD	<LOD
G	0.94	<LOD	<LOD	<LOD	31.85	<LOD	<LOD	<LOD	<LOD	<LOD	<LOD	<LOD	<LOD	<LOD	<LOD
H	<LOD	<LOD	<LOD	<LOD	<LOD	<LOD	<LOD	<LOD	<LOD	<LOD	<LOD	<LOD	<LOD	<LOD	<LOD
I	<LOD	<LOD	<LOD	<LOD	<LOD	<LOD	<LOD	<LOD	<LOD	<LOD	<LOD	<LOD	<LOD	<LOD	<LOD
J	<LOD	<LOD	<LOD	<LOD	<LOD	<LOD	<LOD	<LOD	<LOD	<LOD	<LOD	<LOD	<LOD	<LOD	<LOD
K	<LOD	<LOD	<LOD	<LOD	<LOD	<LOD	<LOD	<LOD	<LOD	<LOD	<LOD	<LOD	<LOD	<LOD	<LOD
L	<LOD	<LOD	<LOD	<LOD	<LOD	<LOD	<LOD	<LOD	<LOD	<LOD	<LOD	<LOD	<LOD	<LOD	<LOD

<LOD (Limit of detection), Concentration in µg kg^−1^.

## Data Availability

The datasets generated for this study are available on request to the corresponding author.

## Supplementary Materials

The following supporting information can be downloaded at: https://www.mdpi.com/article/10.3390/foods12132594/s1, Figure S1: Effect of volume of extraction solvent on extraction efficiency in DLLME. Figure S2: Effect of volumes of disperser solvent on extraction efficiencies in DLLME; Figure S3: Effect of volumes of disperser solvent on extraction efficiencies in DLLME; Figure S4: Chromatogram of mixed contaminants (sulphonamides, fluoroquinolones, pesticides, anthelmintic and aflatoxin B1); Figure S5: Selected ion Chromatograms for individual compounds; Table S1: Recoveries and RSDs of target compounds at different spiked levels in chicken liver using solid phase extraction; Table S2: Physicochemical properties of fluoroquinolones, pesticides, sulphonamides, anthelmintics and aflatoxin B1 [52].
