# Supplementary material for: Simultaneous Determination of Multiple Contaminants in Chicken Liver Using Dispersive Liquid-Liquid Microextraction (DLLME) Detected by LC-HRMS/MS
Source: Foods. 2023 Jul 4;12(13):2594. doi: 10.3390/foods12132594 (PMC10340173; doi:10.3390/foods12132594)
Supplement: Supplementary file 1 [file foods-12-02594-s001.zip › foods-2433105-supplementary.pdf]

Table S1. Recoveries and RSDs of target compounds at different spiked levels in chicken liver using solid phase extraction.

Target Compounds	Added (μg kg $^{-1}$)	Found (μg kg $^{-1}$)	%, Recovery	%, RSD
Aflatoxin B1	2	1.96	98.00	9.01
	5	4.7	94.00	2.47
	20	19.62	98.10	6.95
Albendazole	20	17.28	86.40	3.26
	50	43.64	87.28	2.11
	200	189.37	94.69	4.32
Atrazine	20	19.87	99.35	2.48
	50	44.95	89.90	1.13
	200	196.01	98.01	5.39
Danofloxacin	20	18.01	90.05	7.73
	50	48.83	97.66	9.71
	200	193.54	96.77	8.67
Enrofloxacin	20	16.45	82.25	3.95
	50	46.19	92.38	3.86
	200	183.08	91.54	4.35
Fenbendazole	20	19.09	95.45	7.98
	50	47.71	95.42	7.85
	200	182.57	91.29	4.81
Mebendazole	20	19.18	95.90	3.89
	50	46.69	93.38	5.46
	200	178.23	89.12	7.89
Simazine	20	17.75	88.75	3.04
	50	43.04	86.08	7.48
	200	194.5	97.25	4.35
Sulfachloropyridazine	20	16.74	83.70	8.66
	50	48.84	97.68	8.65
	200	182.75	91.38	8.51
Sulfadiazine	20	19.51	97.55	8.08
	50	45.7	91.40	4.39
	200	190.49	95.25	3.66
Sulfamerazine	20	19.06	95.29	6.39
	50	46.49	92.98	6.39
	200	187.36	93.68	1.95
Sulfaquinoxaline	20	18.53	92.65	2.47
	50	47.16	94.32	9.85
	200	192.34	96.17	6.59
Sulfpyridine	20	16.16	80.80	3.56
	50	46.04	92.08	6.28

Target Compounds	Added (µg kg $^{-1}$)	Found (µg kg $^{-1}$)	%, Recovery	%, RSD
	200	188.15	94.08	4.59
Terbutryn	20	17.75	88.75	9.62
	50	48.67	97.34	3.13
	200	183.53	91.77	5.38
Thiabendazole	20	19.14	95.70	5.56
	50	49.26	98.52	6.39
	200	182.57	91.29	7.83

Table S2. Physicochemical properties of fluoroquinolones, pesticides, sulphonamides, anthelmintics and aflatoxin B1 [52].

Compound	Class	CAS No.	pKa	Log P	Molecular mass
Aflatoxin B1 (AFB1) 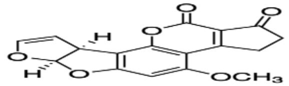	Mycotoxin	1162-65-8	-4.4[a]; 17.79[b]	1.60	312.0634
Albendazole (ABZ) 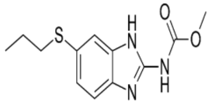	Anthelmintic	54965-21-8	9.51[a]; 4.27[b]	3.22	265.0884
Atrazine (ATZ) 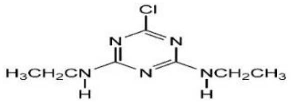	Pesticide	1912-24-9	14.58[a]; 3.38[b]	1.54	215.0938
Danofloxacin (DFX) 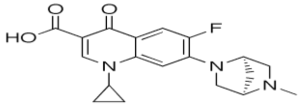	Fluoroquinolone	112398-08-0	5.65[a]; 6.73[b]	0.71	357.1489
Enrofloxacin (ENR) 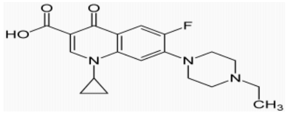	Fluoroquinolone	93106-60-6	5.55[a]; 7.24[b]	-0.58	359.1645
Mebendazole (MEB) 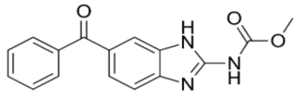	Anthelmintic	31431-39-7	8.44[a]; 3.93[b]	3.26	296.1030
Fenbendazole (FEB)	Anthelmintic	43210-67-9	9.59 [a]; 4.06 [b]	3.39	299.0728

Compound	Class	CAS No.	pKa	Log P	Molecular mass
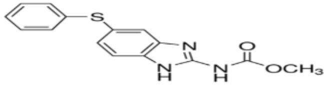					
Simazine (SIZ) 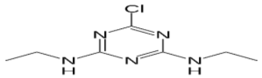	Pesticide	122-34-9	5.28[a]; 7.45[b]	2.3	201.0781
Sulphachloropyridazine (SCP) 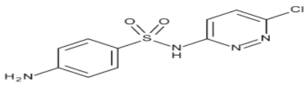	Sulphonamide	803-20-4	6.6[a]; 2.02[b]	0.97	284.0134
Sulphadiazine (SDZ) 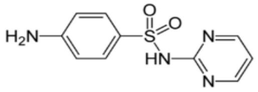	Sulphonamide	68-35-9	6.99[a]; 2.01[b]	0.25	252.0597
Sulphamerazine (SMR) 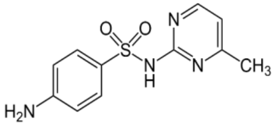	Sulphonamide	127-79-7	6.99[a]; 2.01[b]	0.44	264.0680
Sulphapyridine (SPD) 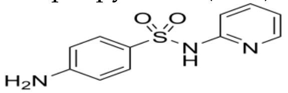	Sulphonamide	144-83-2	6.24[a]; 2.63[b]	0.44	249.0519
Sulphaquinoxaline (SQ) 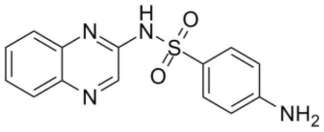	Sulphonamide	967-80-6	6.79 [a]; 2.13 [b]	1.55	300.0681

Compound	Class	CAS No.	pKa	Log P	Molecular mass
Terbutryn (TER) 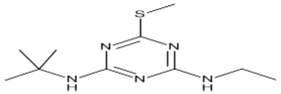	Pesticide	886-50-0	14.31[a]; 6.72[b]	3.65	241.1361
Thiabendazole (TBZ) 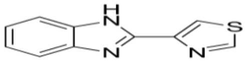	Pesticide	148-79-8	10.28[a]; 4.08[b]	2.47	201.0360

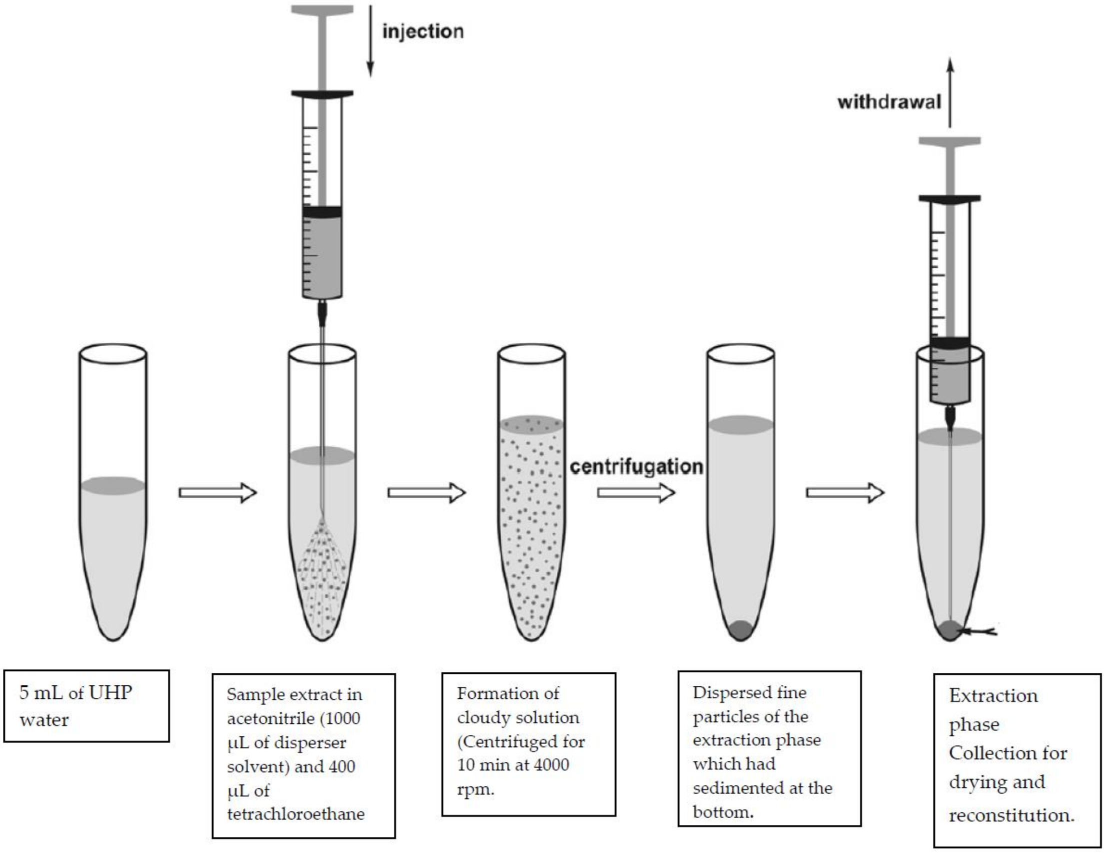

Figure S1. Schematic diagram of the procedure for the extraction of mixed multi-class contaminants [52].

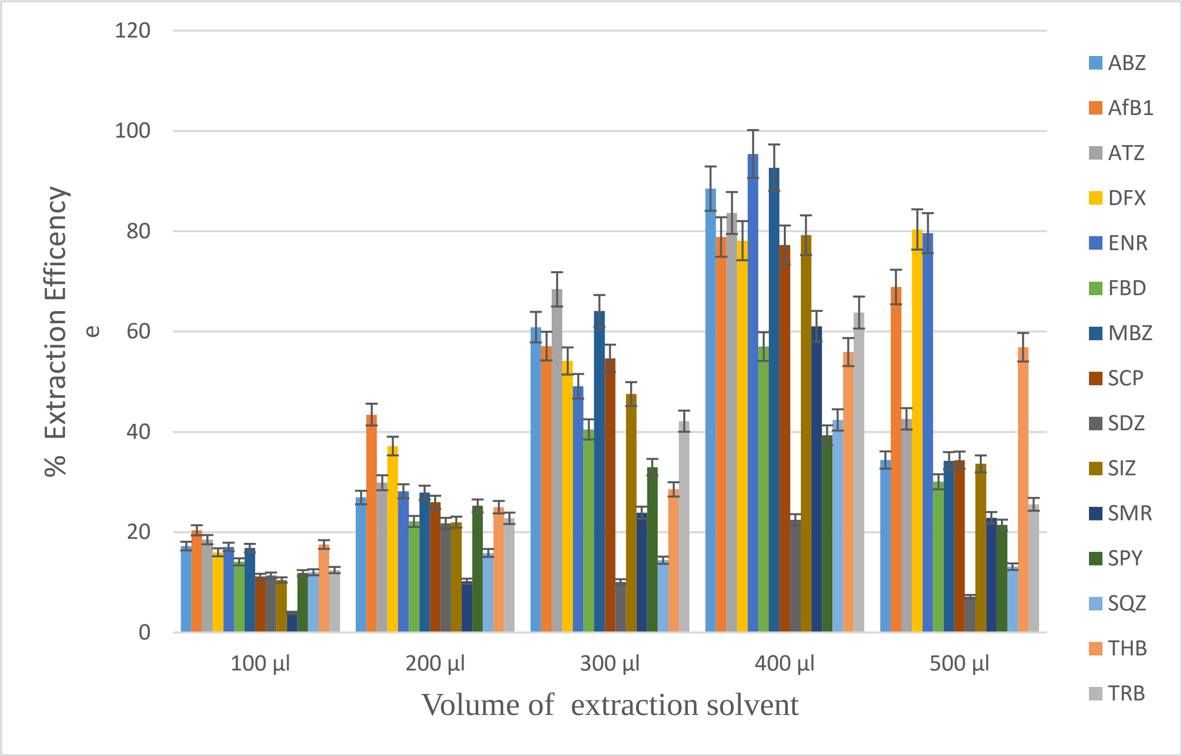

Figure S2. Effect of volume of extraction solvent on extraction efficiency in DLLME. Extraction conditions: 5 mL UHP water; varying volumes (100, 200, 300, 400 and 500 µL) of extraction solvent (tetrachloroethane); 1 000 µL of acetonitrile as disperser solvent; concentration of 100 µg kg^{-1} for sulphonamides, fluoroquinolones, pesticides, anthelmintics; concentration of 10 µg kg^{-1} for aflatoxin B1.

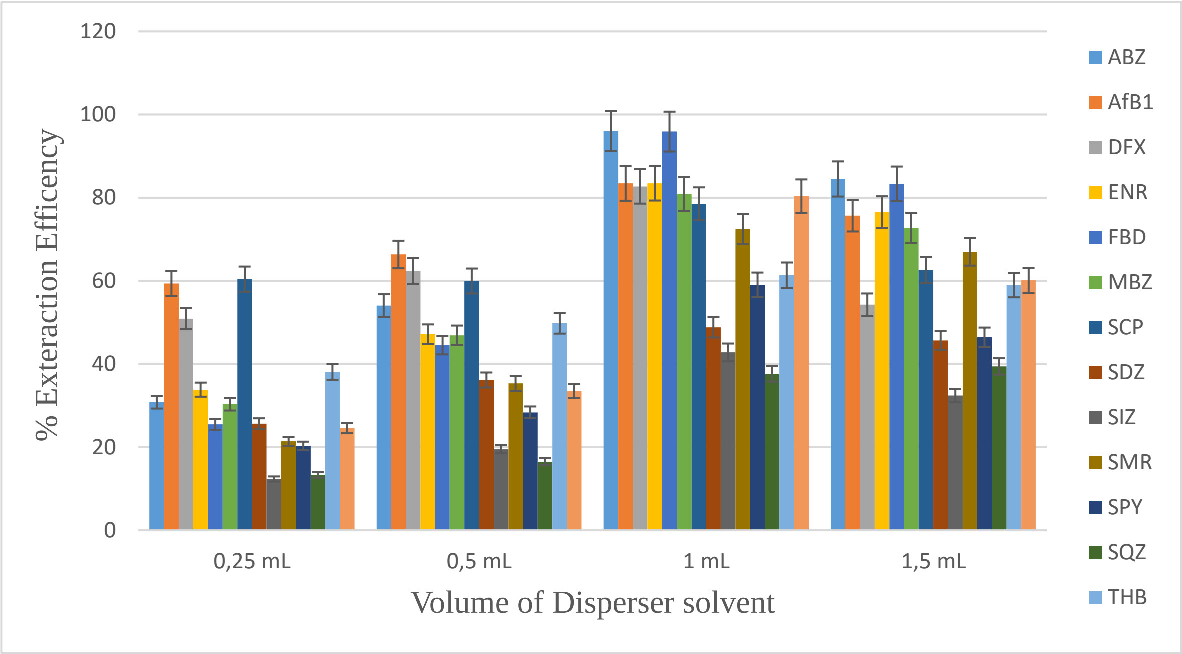

Figure S3. Effect of volumes of disperser solvent on extraction efficiencies in DLLME. Extraction conditions: sample, 5 mL of UHP water; 400 µL of tetrachloroethane as extraction solvent; varying volumes (250, 500, 1 000 and 1 500 µL) of acetonitrile (MeCN) as disperser solvent; concentration of 100 µg kg^{-1} for sulphonamides, fluoroquinolones, pesticides, anthelmintics; concentration of 10 µg kg^{-1} for aflatoxin B1.

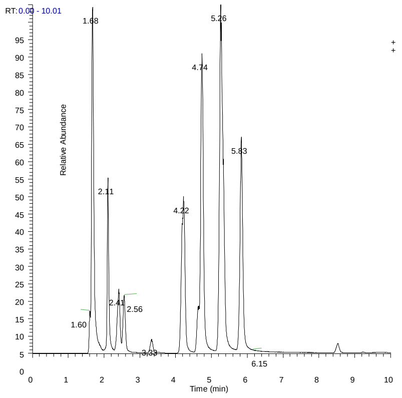

Compound Name	Retention time	Compound Name	Retention time
Aflatoxin B1	4.63	Sulfachloropyridazine	3.33
Albendazole	4.18	Sulfadiazine	2.36
Atrazine	5.83	Sulfamerazine	2.56
Danofloxacin	1.60	Sulfaquinoxaline	4.18
Enrofloxacin	1.68	Sulfpyridine	2.41
Fenbendazole	5.26	Terbutryn	5.28
Mebndazole	4.22	Thiabendazole	2.11
Simazine	4.74		

Figure S4. Chromatogram of mixed contaminants (sulphonamides, fluoroquinolones, pesticides, anthelmintic and aflatoxin B1).

Compound Name	Chromatogram	Mass spec
Aflatoxin B1	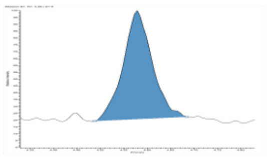	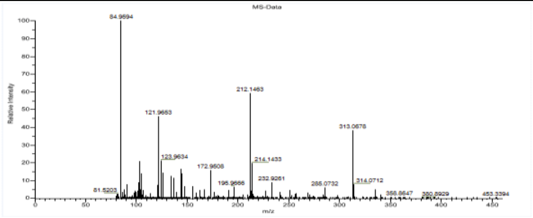
Albendazole	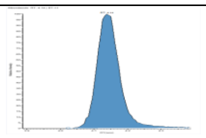	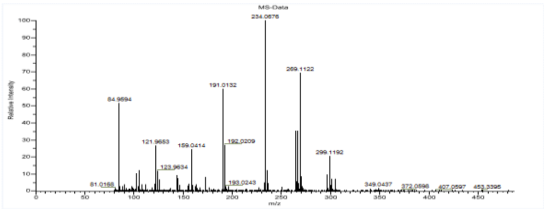
Albendazole-d3	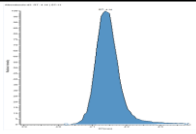	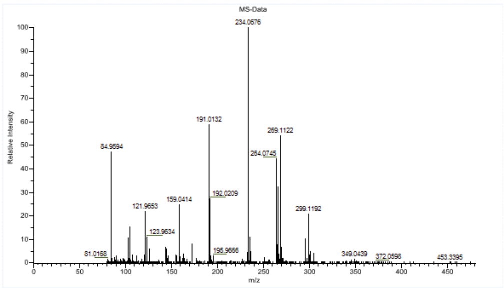
Atrazine	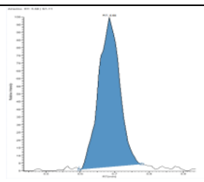	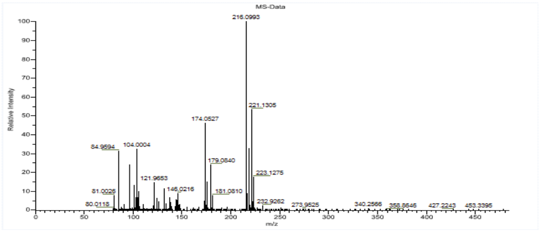
Atrazine -d5	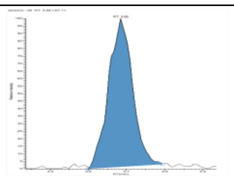	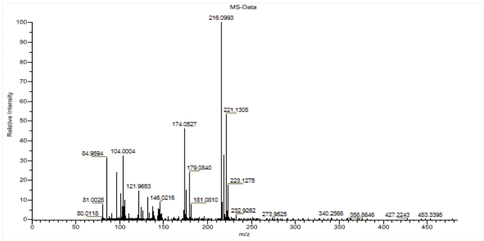
Danofloxacin	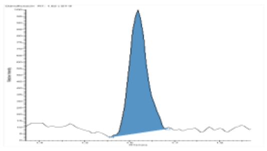	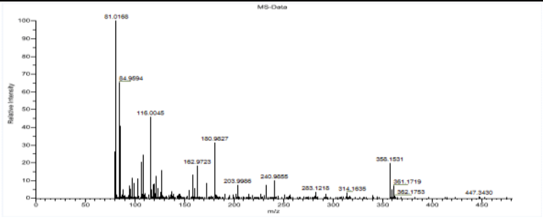
Danofloxacin-(methyl-d3)	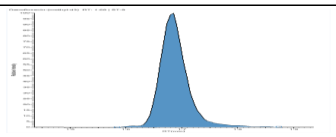	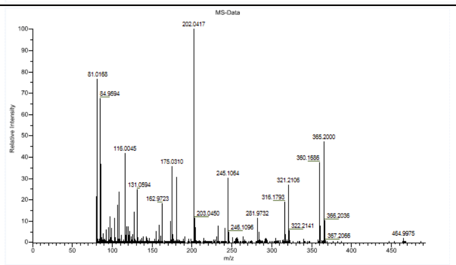

Enrofloxacin	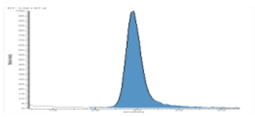	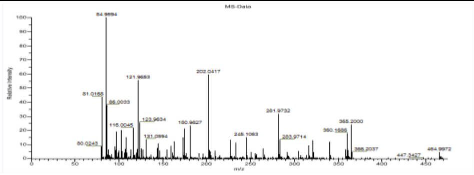
Enrofloxacin-d5	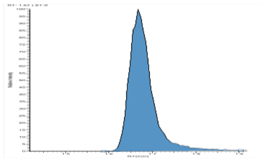	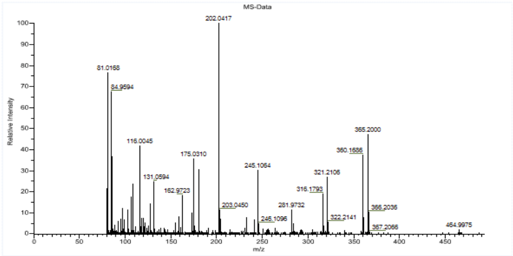
Fenbendazole	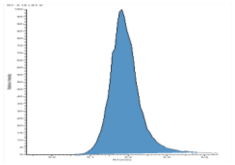	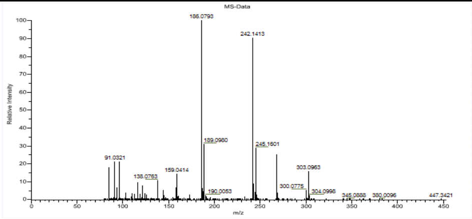
Fenbendazole-d3	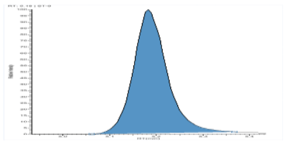	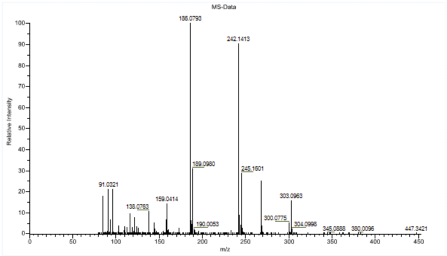
Mebendazole-d3	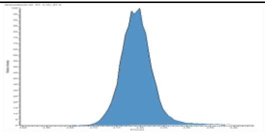	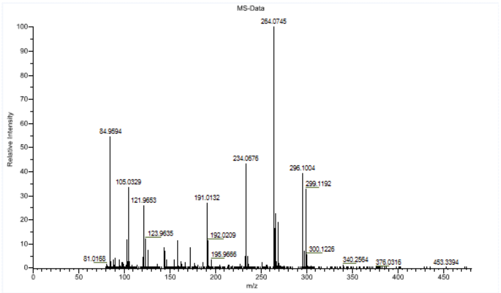
Mebndazole	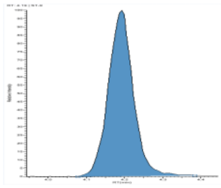	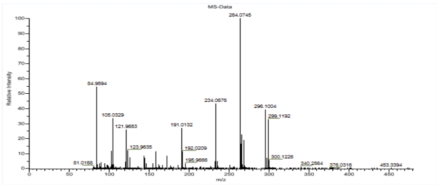
Simazine	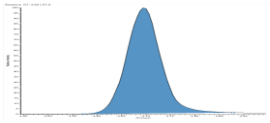	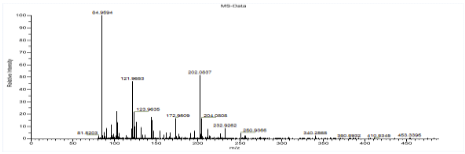

<table>
<tr><td>Simazine-d10</td><td>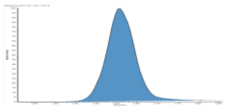</td><td>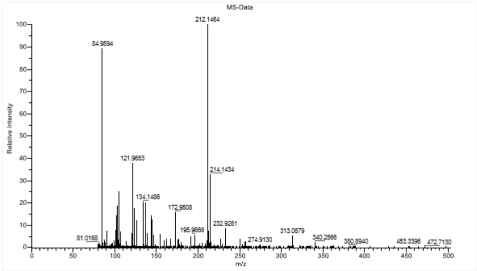
</td></tr>
<tr><td>Sulfachloropyridazine</td><td>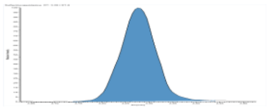</td><td>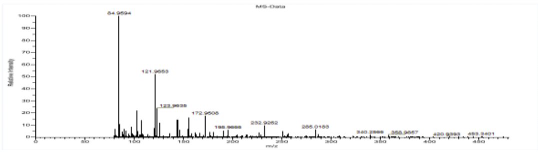
</td></tr>
<tr><td>Sulfadiazine</td><td>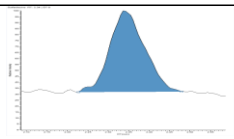</td><td>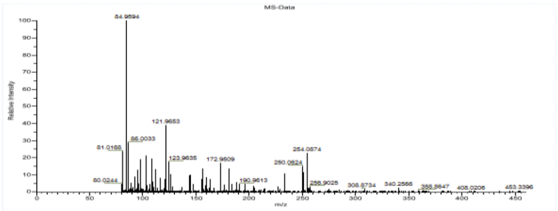
</td></tr>
<tr><td>Sulfamerazine</td><td>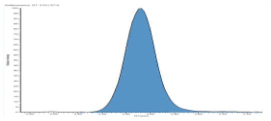</td><td>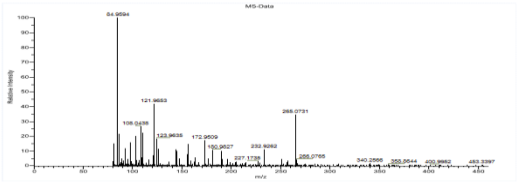
</td></tr>
<tr><td>Sulfamethazine-d4</td><td>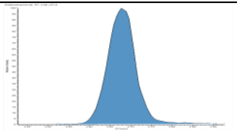</td><td>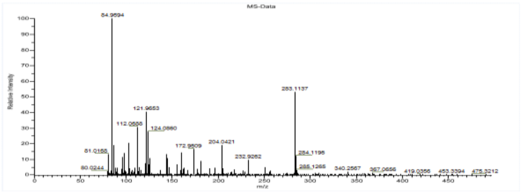
</td></tr>
<tr><td>Sulfaquinoxaline</td><td>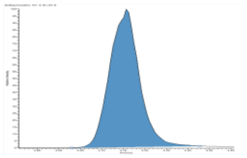</td><td>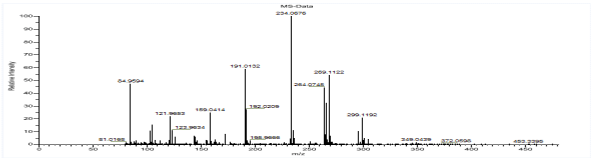
</td></tr>
<tr><td>Sulfaquinoxaline-d4</td><td>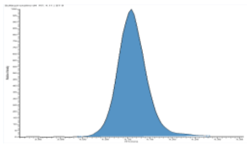</td><td>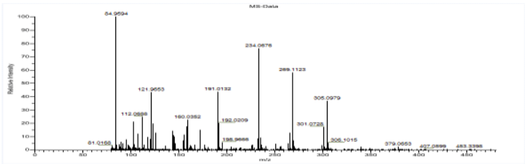
</td></tr>
<tr><td>Sulfpyridine</td><td>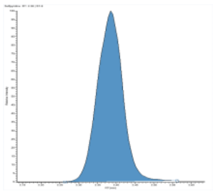</td><td>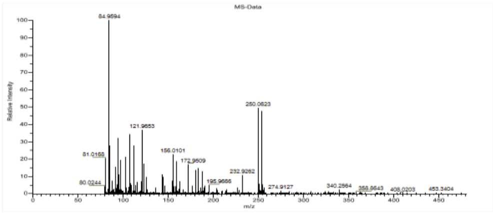
</td></tr>
</table>

Sulfpyridine-d4	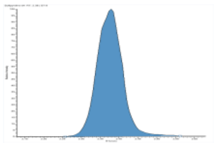	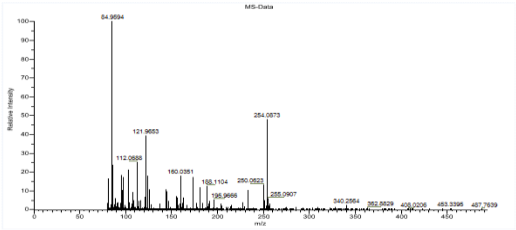
Terbutryn	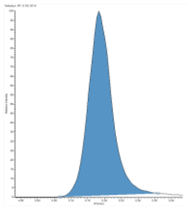	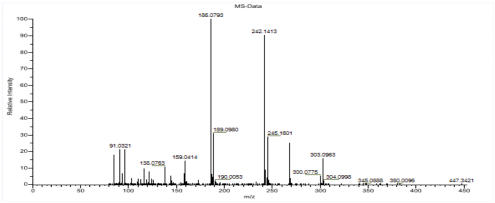
Terbutryn-D5	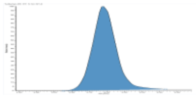	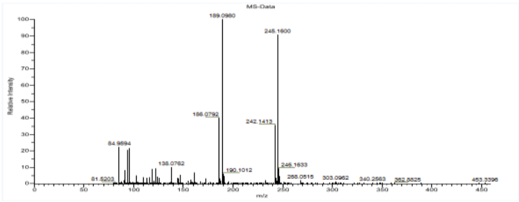
Thiabendazole	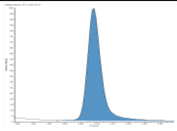	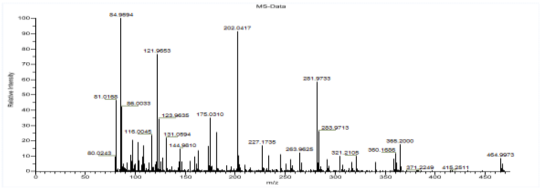

Figure S5. Selected ion Chromatograms for individual compounds.
